# Th17 cells/IL-17A shape *Pasteurella multocida* serotype A infection in murine and rabbit models

**DOI:** 10.1186/s13567-025-01662-1

**Published:** 2025-12-16

**Authors:** Xiongli Liu, Feiyan Lu, Tianci Zhang, Nengzhang Li, Fang He, Yuanyi Peng, Guangfu Zhao

**Affiliations:** 1 College of Veterinary Medicine, Southwest University, Chongqing, China; 2 Department of Endocrinology and Metabolism, Center for Diabetes and Metabolism Research, West China Hospital, Sichuan University, Chengdu, China; 3 College of Animal & Veterinary Sciences, Southwest Minzu University, Chengdu, China

**Keywords:** Pasteurella multocida serotype A, Th17 cells/IL-17A, IL-6–JAK2–STAT3 axis, pathogenesis, T cells, immune response

## Abstract

*Pasteurella multocida* (Pm) is an important zoonotic respiratory pathogen, especially serotype A, which can cause death in various hosts, including humans, farm animals, and wildlife. However, the exact mechanisms by which hosts mobilize the immune system against *Pasteurella multocida* serotype A (PmA) in mammals remain largely unexplored. In the present study, we found that the control of lung infection is critical for PmA infection. Furthermore, unbiased RNA sequencing analysis and histopathological studies revealed that T-cell associated immunity was not fully activated during PmA infection, although the host tended to stimulate Th17 cell and interleukin (IL)-17A-related pathways. To investigate the role of Th17 cells/IL-17A, we employed gain- and loss-of-function Th17 cells and IL-17A at the animal level via inhibitors, through in vitro induction of Th17 cells followed by reperfusion and recombinant IL-17A, respectively. We demonstrated that the host failed to fully activate Th17 cells/IL-17A, which is a key immune mechanism against *P. multocida* infection. Mechanistic studies revealed that the IL-6–JAK2–STAT3 axis contributed to Th17 cell/IL-17A activation in PmA infection. Therefore, using the Stat3 inhibitor Stattic or IL-6KO mouse models significantly limited Th17 cell/IL-17A activation and accelerated host death. Importantly, recombinant IL-17A had strong potential for clinical application, as recombinant IL-17A effectively protected rabbits infected with PmA. Thus, our study revealed that PmA induced T-cell immunodeficiency, and that the activation of Th17 cells/IL-17A via the IL-6–JAK2–STAT3 axis was a key mechanism against PmA. Therefore, targeting Th17 cells/IL-17A could be a potential strategy for the treatment of pasteurellosis and other respiratory bacterial diseases.

**Supplementary Information:**

The online version contains supplementary material available at 10.1186/s13567-025-01662-1.

## Introduction

*Pasteurella multocida* (Pm) is a notorious zoonotic pathogen that causes fatal respiratory illnesses mainly in animals such as rabbits, cattle, and humans [1]. On the basis of the characteristics of bacterial capsular polysaccharides, Pm can be classified into five categories: A, B, D, E, and F [2]. Among them, *Pasteurella multocida* serotype A (PmA) is often found in clinical cases with detectable lung injury [3]. In addition to causing lung injury, PmA can damage the pulmonary epithelial barrier, leading to systemic infections, multiorgan damage, and the release of large amounts of inflammatory factors, ultimately triggering a cytokine storm [4, 5]. Therefore, to control Pm, it is necessary to limit lung infections.

When the host is confronted with a pathogen, the immune system (both innate and acquired) mobilizes its maximum strength to fight the pathogen and minimize damage [6, 7]. However, the immune response is a double-edged sword that needs to be precisely regulated. Inadequate immune responses fail to destroy pathogens, whereas excessive immune responses can cause damage to the host [8]. For pathogens, evading host immunity or inducing inadequate immunity is an important skill to ensure their survival in the host. Although our colleagues and our findings both agree that Pm infection leads to a hyperinflammatory response, the possibility that certain immune cells or inflammatory factors may be suppressed during Pm infection cannot be excluded [4, 9, 10]. Pm has been reported to reduce lymphocyte numbers through apoptosis or cytolysis [11, 12]. *Pasteurella multocida* toxin is considered to manipulate T-cell differentiation [13]. PM27/UPase, purified from Pm, showed significant antiproliferative effects on Concanavalin-A-stimulated mouse spleen cells [14]. These results suggest that Pm may have inhibitory or evasive effects on T-cell associated immunity. In our previous report, based on an unbiased transcriptome analysis, we found that the Th17 cell differentiation-related pathway was enriched in PmA-infected lungs, implying that Th17 cells and their downstream factor IL-17A may be involved in PmA infection [15].

Th17 cells were first recognized as a distinct subpopulation of Th0 cells in 2005 [16]. These cells originate from naïve CD4^+^ T cells and develop under the guidance of antigen-presenting cells (APCs) and the cytokines they secrete. Th17 cells are known for their specific secretion of interleukin (IL)-17, which plays a key role in triggering tissue inflammation [16, 17]. Th17 cells have been reported to be critical for antifungal responses and host defense against bacterial infections (extracellular) [18]. IL-17A, the most representative member of the IL-17 family, is a key product of Th17 cells and has been shown to be a key cytokine in the protection of the host against mucosal infections through its receptor [19]. However, excessive numbers of Th17 cells or IL-17A can induce autoimmune diseases and promote infections [20–22]. Furthermore, Th17 cells and IL-17A promote lung inflammation and injury [22–24]. Therefore, clarifying whether Th17 cells/IL-17A exert protective or detrimental effects during infections is essential for effectively controlling disease progression. Currently, no study has examined whether Th17 cells/IL-17A are fully activated during Pm infection, and the role of Th17 cells/IL-17A in Pm infection has not been clarified. Therefore, on the basis of our previous study [15], we further systematically explored the role of Th17 cells/IL-17A in pasteurellosis.

Here, we demonstrated that Th17 cell/IL-17A activation is involved in the pathogenesis of PmA in mammals on the basis of unbiased RNA sequencing and histopathological techniques. Furthermore, we demonstrated that the host failed to induce sufficient Th17 cells/IL-17A during PmA infection, as evidenced by the significant reduction in Pm-induced mortality following exogenous supplementation of Th17 cells/IL-17A. At the molecular level, using a knockout mouse model, we found that the IL-6–JAK2–STAT3 axis played an important role in Th17 cell/IL-17A activation during PmA infection. Our findings suggested that targeting the Th17 cell/IL-17A activation pathway may be a potential strategy for the treatment of pasteurellosis and other respiratory bacterial diseases.

## Materials and methods

### Bacterial strains and culture conditions

Bovine *Pasteurella multocida* PmCQ2 (serotype A; GenBank no. CP033599) and PmCQ6 (serotype A; GenBank no. CP033600) strains were stored in our laboratory at 1-week LD50 values of 1 colony forming units (CFU) and 10^8^ CFU, respectively. All *Pasteurella multocida* strains were cultured on Martin’s broth agar with 5% horse serum at 37 °C.

### Animal experiments

All animal experiments followed the National Research Council’s Guide for the Care and Use of Laboratory Animals (8th edition). The experimental protocols were approved by the Institutional Animal Care and Use Committee (IACUC) of Southwest University, China (no. LAC2024-1-0067). Eight-week-old male CD-1 (ICR) mice weighing 30–35 g were obtained from HFK Bioscience (Beijing, China), and 2-month-old male New Zealand rabbits weighing 1500 g were obtained from Enswell Biotech (Chongqing, China). IL-6KO mice were purchased from Shanghai Model Organisms Center, Inc., and IL-6KO mice were characterized via PCR (Additional file 4). The animals were housed in individual IVC rabbit cages with a 12 h light/dark cycle, the temperature was maintained at 25 °C, and the animals had free access to food and water. The animals were randomly assigned to each experimental group.

For infection experiments, animals were anesthetized with 1.5% pentobarbital sodium and intranasally infected with 10^4^ CFU PmCQ2 (mice), 10^9^ CFU PmCQ2 (rabbits), or 10^7^ CFU PmCQ6. Following the schematic in the Results section, the mice received Stattic (MCE, HY-13818), recombinant mouse IL-17A (MCE, HY-P73174), recombinant rabbit IL-17A (MCE, HY-P73175), bovine serum albumin (BSA; BioFroxx, 4240GR100), an anti-mouse IL-17A-living antibody (Selleck, A2120), a rat immunoglobulin G1 (IgG1) isotype control-living antibody (Selleck, A2119), or saline. After completion of the animal experiments, the mice were euthanized under 1.5% sodium pentobarbital anesthesia for blood and tissue collection.

### Histological and multiplex immunohistochemical (mIHC) examinations

The collected tissues were immediately fixed with 4% paraformaldehyde (*w*/*v*), embedded in paraffin, and sectioned into 5-µm thick sections. Hematoxylin‒eosin (HE) was applied according to the manufacturer’s instructions (Beyotime Biotechnology, C0105M). Multiplex immunohistochemistry (mIHC) was performed according to the manufacturer’s instructions (Zenbio, 18 002). Briefly, the slides were deparaffinized, rehydrated, and retrieved. Then, the slides were treated with 3% H_2_O_2_ and blocked with 5% bovine serum albumin. The slides were incubated with primary antibodies against IL-17A (Proteintech, 66 148-1-Ig), CD3 (ABclonal, A19017), CD4 (Proteintech, 19 068-1-AP), and CD68 (Zenbio, 501 199), followed by incubation with the corresponding secondary antibodies conjugated to horseradish peroxidase (HRP) and corresponding TSA(Tyramide signal amplification) dye. For the second primary antibody, repeated retrieval, blocking, primary antibody incubation, secondary antibody incubation, and TSA staining were performed. Finally, the slides were stained with DAPI and visualized via fluorescence microscopy.

### Quantitative real‑time PCR

Total RNA was extracted via the AFTSpin Tissue/Cell Fast RNA Extraction Kit for Animals (ABclonal, RK30120) and subsequently reverse transcribed into a cDNA pool with ABScript III RT Master Mix for qPCR, which includes gDNA Remover (ABclonal, RK20429). Real-time PCR was conducted with a Bio-Rad CFX96 instrument. The relative mRNA expression levels were normalized to those of beta-actin. The complete sequences of the real-time PCR primers are provided in Additional file 5.

### RNA sequencing

The RNA sequencing analysis was conducted by OE Biotech, Inc., located in Shanghai, China. In summary, mouse lung tissues were collected 24 h after infection and subsequently homogenized in liquid nitrogen. Total RNA extraction was performed via TRIzol reagent. The ribosomal RNA (rRNA) was eliminated with the Ribo-Zero rRNA Removal Kit (Illumina, MRZH116). For each sample, a total of 1 μg of RNA was used to construct the cDNA library. The quality and size of the cDNA library were evaluated via an Agilent 2100 Bioanalyzer. Ultimately, the Illumina HiSeq 4000 platform was employed to generate 150 bp paired-end reads from the cDNA sequences. The raw RNA sequencing data can be found in Additional file 6 and NCBI (ID: PRJNA1330503).

### Western blotting

Tissues were lysed via RIPA buffer supplemented with a protease inhibitor cocktail, and homogenization was performed as part of the process. Following centrifugation at 4 °C, the protein in the supernatant was collected. The protein concentration of the cell lysates was determined via the bicinchoninic acid (BCA) assay (Thermo Fisher, cat. no. 23 227), after which the samples were boiled in the presence of a reducing agent (DTT). Proteins were separated by sodium dodecyl sulfate–polyacrylamide gel electrophoresis (SDS‒PAGE), transferred onto PVDF membranes, and blocked with 5% nonfat milk. The membranes were then incubated with primary antibodies against p-STAT3 (Cell Signaling Technology, 9145), STAT3 (ABclonal, A22434), p-JAK2 (Zenbio, R381556), JAK2 (Zenbio, R24775), and IL-17A (Proteintech, 66 148–1-Ig). The membranes were subsequently treated with HRP-conjugated secondary antibodies (Boster Bio, BA1054, BA1050, and BA1060) and visualized via an enhanced chemiluminescence (ECL) detection kit (Bio-Rad, cat. no. 170-5060).

### Flow cytometry

Th17 cells were identified via a Mouse Th17 Cell Flow Cytometry Staining Kit (Elabscience, XJM002). Briefly, mouse lungs were prepared as single-cell suspensions, resuspended in ACK lysis buffer (Elabscience, E-CK-A105) for 5 min, and resuspended in RPMI-1640 medium. The cells were adjusted to 10^6^ cells per group and incubated with the Cell Stimulation Mix and Protein Transport Inhibitor under 5% CO_2_ at 37 °C for 5 h. The cells were subsequently resuspended in cell staining buffer, fixed in fixation buffer, permeabilized in permeabilization buffer, and incubated with antibodies (PerCP/cyanine5.5–CD3, FITC–CD4, and PE–IL-17A) at room temperature for 60 min. Finally, the stained Th17 cells were detected via flow cytometry. The gating strategy for the identification of Th17 cells in mouse lungs is provided in Additional file 3.

### In vitro Th17 cell differentiation

In vitro Th17 cell differentiation was performed according to a previous study with slight modifications [25]. Briefly, naïve CD4^+^ T cells were isolated from mouse spleens via naïve CD4^+^ T-cell isolation kits (STEMCELL, 19765). Naïve CD4^+^ T cells (1 × 10^5^ cells) were cultured in a 96-well plate with anti-mouse CD3 (2.5 μg/mL) (Multi Science, F2100300) and anti-mouse CD28 (2 μg/mL) (Multi Science, F2102800) antibodies. For Th17 cell differentiation, T cells were cultured with 10 ng/mL recombinant TGF-β1 (ABclonal, RP00671), 80 ng/mL recombinant IL-6 (ABclonal, RP01321), 20 ng/mL recombinant IL-23 (ABclonal, RP01160), 10 ng/mL recombinant TNF-α (ABclonal, RP01071), or 10 ng/mL recombinant IL-1β (ABclonal, RP01340) for 3 days. For the identification of Th17 cells, cultured Th17 cells were treated with 50 ng/mL PMA, 750 ng/mL ionomycin, and 10 μg/mL brefeldin (Elabscience, E-CK-A091) to induce IL-17A, and positive Th17 cells were identified via flow cytometry (Additional file 3).

### ELISA and detection of biochemical indices

The concentrations of mouse IL-17A (Invitrogen, 88-7371-88), IL-1β (Invitrogen, 88-7013A-88), IL-6 (Invitrogen, 88-7064-88), and TNF-α (Invitrogen, 88-7324-88) in mouse serum were determined via ELISA, as per the manufacturer’s guidelines. The concentrations of mouse aspartate aminotransferase (AST; Solarbio, BC1565), alanine aminotransferase (ALT; Solarbio, BC1555), blood urea nitrogen (BUN; Solarbio, BC1535), and creatinine (Elabscience, E-BC-K188-M) in mouse serum were determined via ELISA, as per the manufacturer’s guidelines.

### Statistical analysis

Statistical analyses were performed via GraphPad Prism (version 6.0) and PASW Statistical 18.0 (SPSS). The data were grouped according to the mean ± standard deviation (SD) to summarize distinct datasets. The significance of differences among the data were evaluated via two-tailed Student’s *t*-tests, Mann‒Whitney *U* tests, or one-way analysis of variance (ANOVA), as appropriate. Survival rates were assessed via a log-rank (Mantel‒Cox) test following the construction of Kaplan‒Meier curves. Statistical significance levels are indicated as follows: *p < 0.05, **p < 0.01, and ***p < 0.001.

## Results

### The ability to destroy the pulmonary barrier is crucial for the pathogenicity of *Pasteurella multocida* serotype A

**Figure 1:**
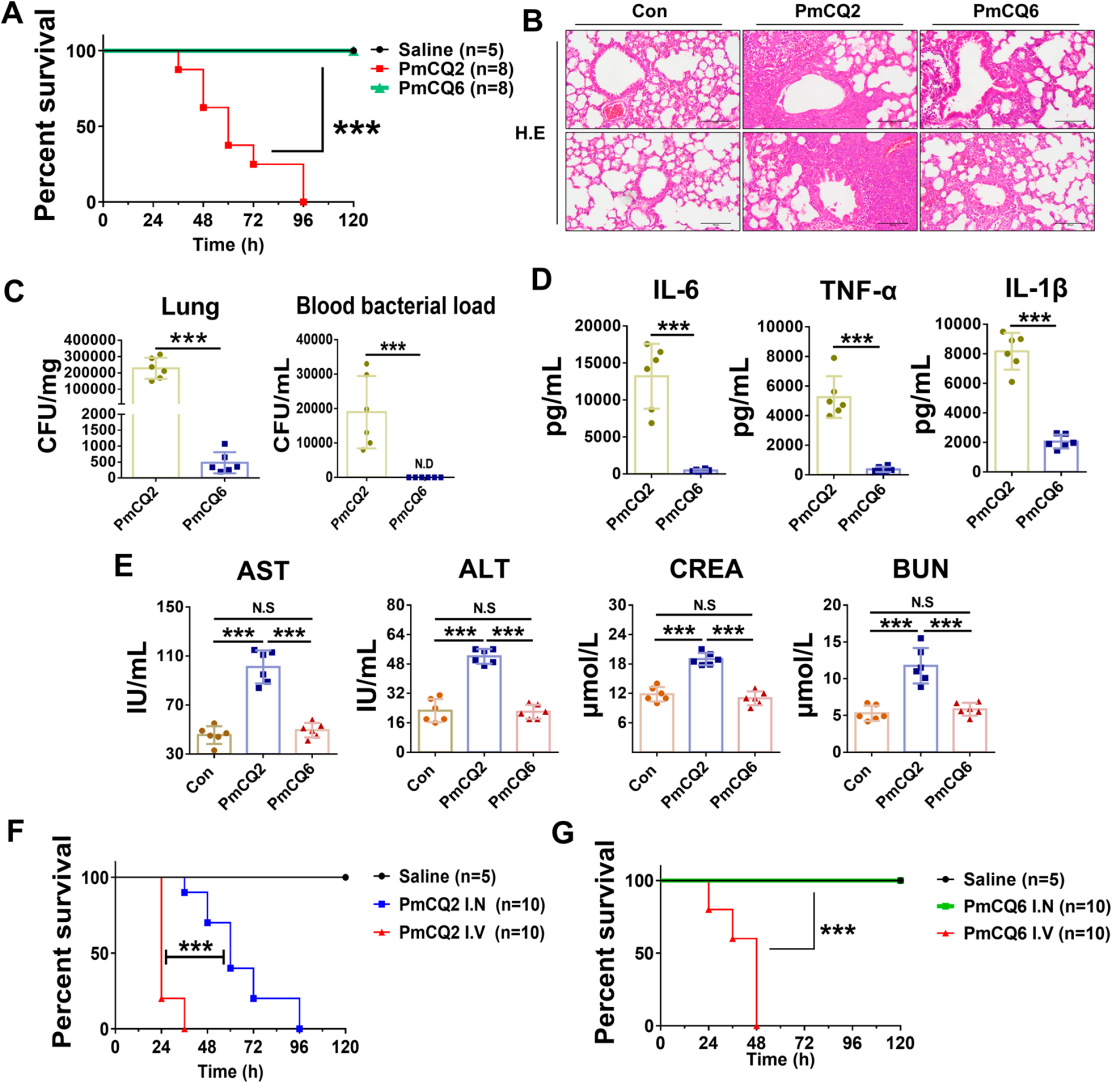
**The ability to destroy the pulmonary barrier is crucial for the pathogenicity of** ***Pasteurella multocida*** **serotype A.** **A** Survival curves of mice infected with saline, PmCQ2, or PmCQ6. **B** Representative images of HE staining of murine lungs infected with saline, PmCQ2, or PmCQ6 at 32 hpi (hour post-infection). Scale bars = 200 μm. HE staining revealed that compared with PmCQ6 infection, PmCQ2 infection significantly exacerbated pulmonary injury and inflammatory response, accompanied by thickening of alveolar walls and visible deposits of red blood cells, inflammatory cells, and fibrin within the alveolar spaces. **C** The bacterial loads of PmCQ2- and PmCQ6-infected lungs and blood at 32 hpi. **D** Quantification of IL-6, TNF-α, and IL-1β in PmCQ2- and PmCQ6-infected murine serum at 32 hpi. **E** Quantification of aspartate aminotransferase (AST), alanine aminotransferase (ALT), urea nitrogen (BUN), and creatinine (CREA) levels in PmCQ2- and PmCQ6-infected murine serum at 32 hpi. **F** Survival curves of Con (saline) group mice, intranasal PmCQ2-infected group mice, and intravenous PmCQ2-infected group mice. **G** Survival curves of Con (saline) group mice, intranasal PmCQ6-infected group mice, and intravenous PmCQ6-infected group mice. Every point represents one individual. N.S., not significant; ***p < 0.001.

Consistent with our previous report [4], intranasal infection with high virulence PmCQ2 resulted in the death of mice, whereas intranasal infection with low virulence PmCQ6 did not (Figure 1A). HE staining showed that PmCQ2 induced more severe lung lesions than PmCQ6 did (Figure 1B). The bacterial loads of the infected lungs and blood were also significantly greater with PmCQ2 than with PmCQ6 (Figure 1C). This finding suggested that PmCQ2 was more difficult to clear by the host and disrupted the lung barrier. Consequently, we found that systemic inflammation and extrapulmonary impairments were much more severe in PmCQ2-infected mice than in PmCQ6-infected mice, as evidenced by the upregulation of IL-6, TNF-α, and IL-1β, as well as the biochemical indices of hepatic and renal functions, such as aspartate aminotransferase (AST), alanine aminotransferase (ALT), blood urea nitrogen (BUN), and creatinine (Figures 1D, E). In addition, we artificially injected PmCQ2 and PmCQ6 into the bloodstream of mice via intravenous injection and found that either PmCQ2 or PmCQ6 significantly increased their pathogenicity, which is also consistent with the findings of our previous study [4] (Figures 1F, G). These data demonstrate that bloodstream invasion by PmA following pulmonary barrier breach is a key determinant of virulence. In other words, if PmA can be effectively cleared at the lung infection stage, its pathogenicity to the host is significantly reduced.

### Transcriptomic data suggest that Th17 cell/IL-17A-related signaling is involved in the *Pasteurella multocida* serotype A infection

**Figure 2:**
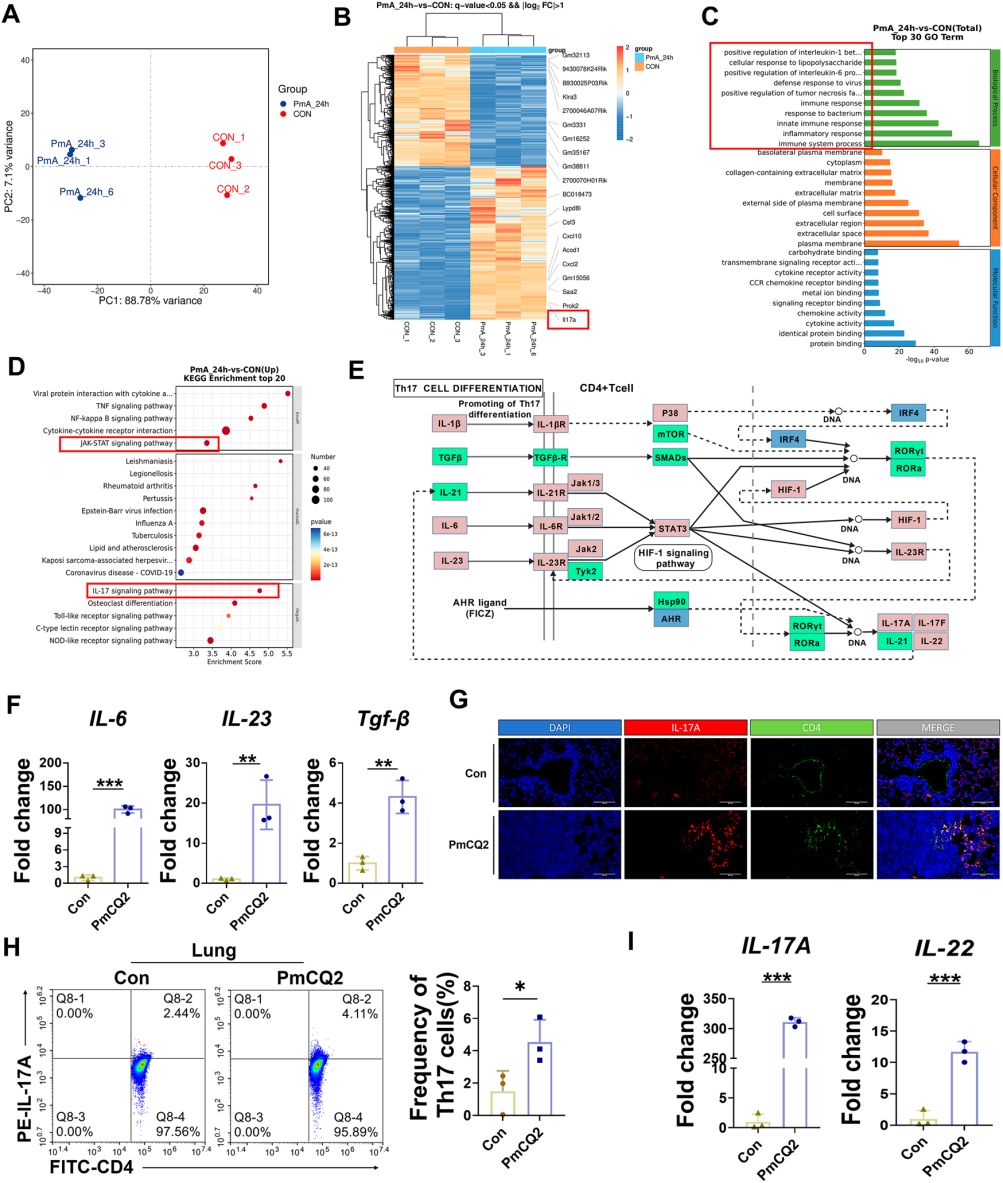
**Transcriptomic data suggest that Th17 cell/IL-17A-related signaling is involved in the *Pasteurella multocida*** **serotype A infection.** A Principal component analysis (PCA) of the transcriptomic data of the PmCQ2 groups and Con groups. **B** Transcriptomic analysis of significantly different genes between the PmCQ2 groups and Con groups. **C** Gene Ontology (GO) analysis of PmCQ2 groups versus Con groups and the top 30 GO terms are shown. **D** Kyoto Encyclopedia of Genes and Genomes (KEGG) analysis of PmCQ2 groups versus Con groups; the top 20 pathways are listed. **E** Th17 cell differentiation-associated genes identified via KEGG are shown. Red indicates upregulation and blue indicates downregulation. **F** Transcription levels of IL-6, IL-23, and TGF-β in PmCQ2-infected murine lungs at 24 hpi. **G** Representative images of mIHC staining of IL-17A and CD4 in Con- and PmCQ2-infected murine lungs at 32 hpi. The double-positive cells were mature Th17 cells. Scale bar = 100 μm. **H** Flow cytometry analysis of Th17 cells in PmCQ2-infected murine lungs at 32 hpi. (Right) quantification of Th17 cells in PmCQ2-infected murine lungs at 32 hpi. **I** Transcription levels of IL-17A and IL-22 in PmCQ2-infected murine lungs at 24 hpi. Every point represents one individual. *p < 0.05, **p < 0.01, ***p < 0.001.

To elucidate why the host failed to efficiently clear PmCQ2 from the lungs, we performed comprehensive transcriptome analysis. As shown in Figures 2A, B, transcript levels were significantly altered in PmCQ2-infected lungs compared with those in wild-type lungs. Gene Ontology (GO) analysis revealed that the immune system was significantly affected by PmCQ2 (Figure 2C). We noted that the IL-17 signaling pathway and the JAK–STAT pathway were enriched in the Kyoto Encyclopedia of Genes and Genomes (KEGG) analysis (Figure 2D), suggesting that the Th17 cell/IL-17A signaling pathway is important in PmCQ2 infection. Furthermore, genes involved in Th17 cell/IL-17A maturation were significantly upregulated during PmCQ2 infection, suggesting that Th17 cells/IL-17A may play a key role in PmA infection (Figure 2E). We subsequently demonstrated that three key molecules that promote Th17 cell differentiation were significantly upregulated during PmCQ2 infection (Figure 2F) and verified that PmA induced mature Th17 cells in situ in the lungs of PmA-infected mice via various methods, including immunohistochemistry and flow cytometry (Figures 2G, H). We also found that mature Th17 cells were induced in situ in the lungs of PmA-infected rabbits (Additional file 1). In addition, the transcription of the Th17 cell effector cytokines IL-17A and IL-22 was correspondingly upregulated in PmCQ2-infected mouse lungs (Figure 2I). These results suggested that Th17 cell/IL-17A-related signaling is involved in the process of PmA infection.

### Th17 cells inhibit *Pasteurella multocida* serotype A infection

**Figure 3:**
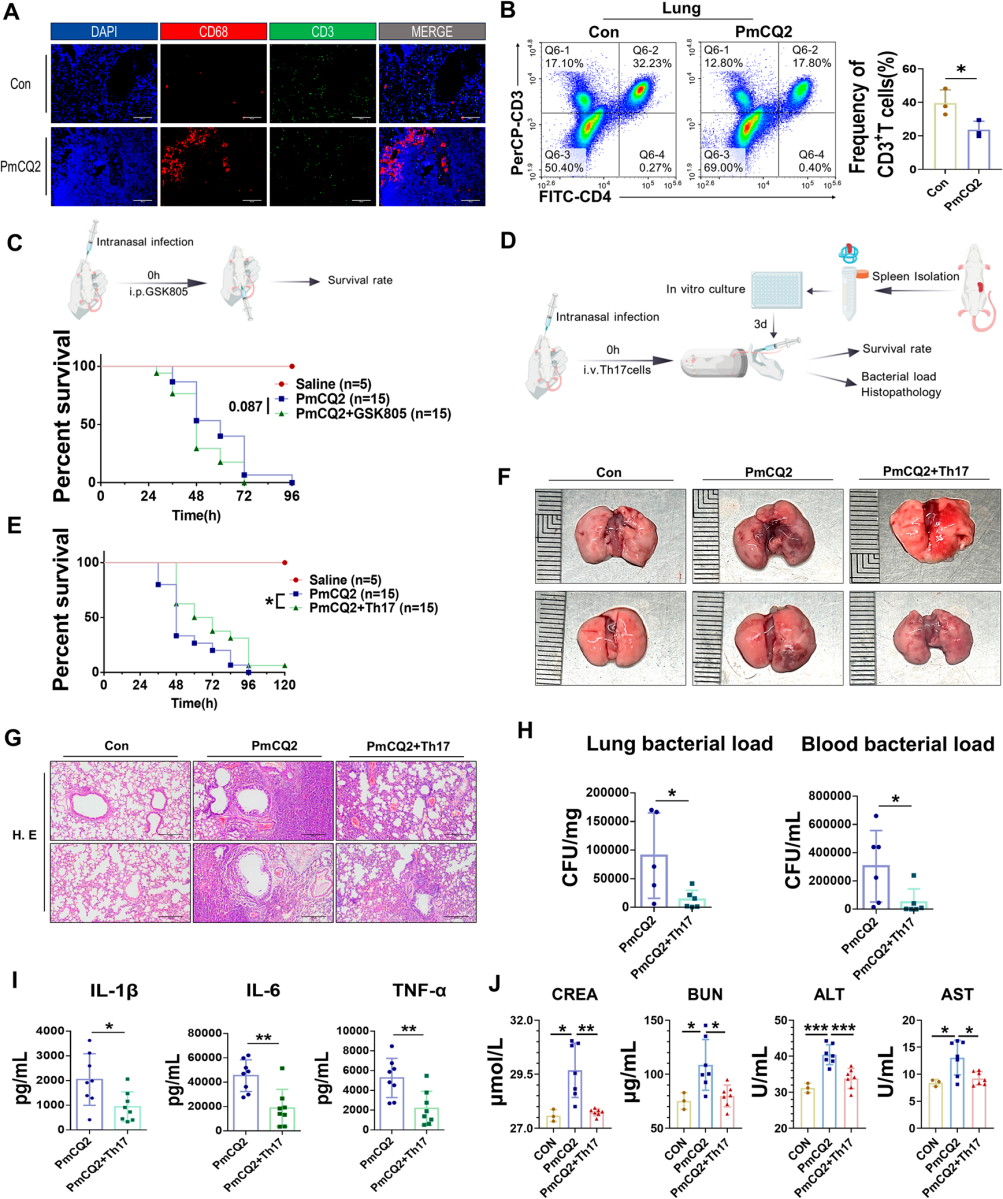
**Th17 cells inhibit *Pasteurella multocida* serotype A infection.** **A** Representative images of mIHC staining of the macrophage marker CD68 and the T-cell marker CD3 in Con- and PmCQ2-infected murine lungs at 32 hpi. Scale bar = 100 μm. **B** Flow cytometry analysis and quantification of CD3^+^ T cells in Con- and PmCQ2-infected murine lungs at 32 hpi. **C** A scheme showing the GSK805 (30 mg/kg) treatment protocol at the top. The survival curves of PmCQ2-infected mice treated with or without 30 mg/kg GSK805 are shown at the bottom. **D** A scheme presents the in vitro Th17 cell differentiation and treatment assay protocol. **E** Survival curves of PmCQ2-infected mice treated with or without 10^6^ Th17 cells. **F** Representative photographs of murine lungs infected with PmCQ2 plus saline or Th17 cells at 32 hpi. **G** Representative images of HE staining of murine lungs infected with PmCQ2 plus saline or Th17 cells at 32 hpi. Scale bar = 200 μm. **H** The bacterial load of the infected lungs and blood at 32 hpi. **I** Quantification of IL-6, TNF-α, and IL-1β PmCQ2-infected murine serum at 32 hpi. **J** Quantification of AST, ALT, BUN, and CREA levels in PmCQ2-infected murine serum at 32 hpi. Every point represents one individual. *p < 0.05, **p < 0.01, ***p < 0.001.

Considering that the activation and function of Th17 cells are involved in T-cell immunity, we counted T cells in the lungs of mice with CD3^+^ T cells. The results revealed that the expression of CD3 in the lungs of PmCQ2-infected mice and rabbits did not increase significantly, but rather tended to decrease (Figures 3A, B; Additional file 2), which is in agreement with related reports [11, 12, 14]. CD3 is not only a T-cell marker but also a key activation molecule for T cells. Therefore, we hypothesized that PmA suppressed T-cell immunity and that Th17 cells may be key to host resistance to PmA. As shown in Figure 3C, the commercially available Th17 cell inhibitor GSK805 indeed promoted PmA-induced death, suggesting that Th17 cells play a role in PmA infection. Furthermore, to further assess the functional competence of host Th17 cells, we differentiated murine naïve CD4^+^ T cells into Th17 cells and infused them into PmA-infected mice (Figure 3D; Additional file 3). As shown in Figures 3E–G, receiving additional Th17 cells significantly increased the survival rate and reduced the lung lesions in PmA-infected mice. Correspondingly, the bacterial load in the lungs and blood, systemic inflammation, and extrapulmonary injury were also significantly reduced after additional Th17 cells were administered (Figures 3H–J). These results suggested that PmA limits T-cell immunity while revealing a critical protective function of Th17 cells during infection.

### IL-17A, a Th17 cell effector, restricts *Pasteurella multocida* serotype A infection

**Figure 4:**
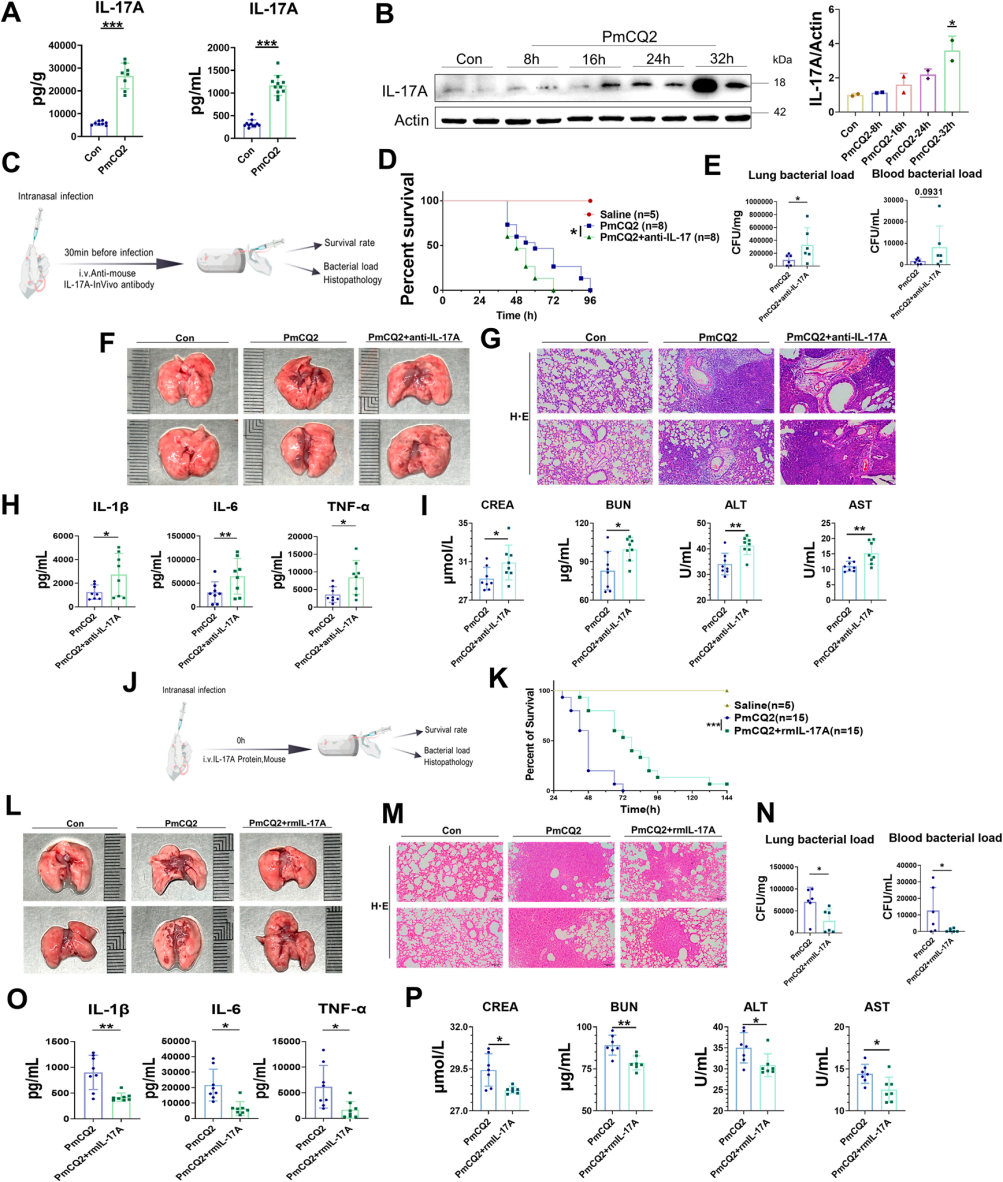
**IL-17A, a Th17 cell effector, restricts *Pasteurella multocida* serotype A infection.** **A** Quantification of IL-17A in PmCQ2-infected murine blood and lungs at 32 hpi. **B** Western blot analysis and quantification of IL-17A in PmCQ2-infected murine lungs. **C** A schematic of the mouse IL-17A neutralizing antibody treatment protocol. **D** Survival curves of PmCQ2-infected mice treated with or without 200 μg of mouse IL-17A neutralizing antibody. **E** The bacterial load of the infected lungs and blood at 32 hpi. **F** Representative photographs of murine lungs infected with PmCQ2 plus IgG or 200 μg of mouse IL-17A neutralizing antibody at 32 hpi. **G** Representative images of HE staining of murine lungs infected with PmCQ2 plus IgG or 200 μg of mouse IL-17A neutralizing antibody at 32 hpi. Scale bar = 200 μm. **H** Quantification of IL-6, TNF-α, and IL-1β PmCQ2-infected murine serum at 32 hpi. **I** Quantification of AST, ALT, BUN, and CREA levels in PmCQ2-infected murine serum at 32 hpi. J A schematic of the 10 μg/kg recombinant mouse IL-17A treatment protocol. **K** Survival curves of PmCQ2-infected mice treated with or without 10 μg/kg recombinant mouse IL-17A. **L** Representative photographs of murine lungs infected with PmCQ2 plus 10 μg/kg BSA or 10 μg/kg recombinant mouse IL-17A at 32 hpi. **M** Representative images of HE staining of murine lungs infected with PmCQ2 plus 10 μg/kg BSA or 10 μg/kg recombinant mouse IL-17A at 32 hpi. Scale bar = 200 μm. **N** The bacterial load of the infected lungs and blood at 32 hpi. **O** Quantification of IL-6, TNF-α, and IL-1β PmCQ2-infected murine serum at 32 hpi. **P** Quantification of AST, ALT, BUN, and CREA levels in PmCQ2-infected murine serum at 32 hpi. Every point represents one individual. *p < 0.05, **p < 0.01, ***p < 0.001.

Th17 cells are named because they secrete IL-17 [26]. IL-17A, the most representative member of the IL-17 family, has been reported to be bifacial in lung injury [23]. During PmA infection, IL-17A was significantly increased in PmCQ2-infected lungs and blood, as determined via ELISA and western blot analyses (Figures 4A, B). To investigate the role of IL-17A during PmA infection, the mice were supplemented with IL-17A-neutralizing antibodies (Figure 4C). As shown in Figure 4D, supplementation with an anti-IL-17A neutralizing antibody significantly reduced the survival of PmCQ2-infected mice. IL-17A neutralizing antibodies also exacerbated lung injury, which was consistent with an increase in the bacterial load in the infected lungs and in the bloodstream (Figures 4E–G). Correspondingly, we found that systemic inflammation and extrapulmonary injury were more severe after IL-17A-neutralizing antibody treatment (Figures 4H, I), as evidenced by the upregulation of IL-6, TNF-α, and IL-1β, as well as biochemical markers of hepatic and renal function (AST, ALT, BUN, and CREA). These results suggested that IL-17A played an important role in the fight against PmA infection. To determine whether the host produced sufficient IL-17A, we injected PmA-infected mice with additional recombinant IL-17A (Figure 4J). In contrast to IL-17A-neutralizing antibodies, additional injections of recombinant IL-17A significantly increased the survival of PmA-infected mice, and recombinant IL-17A also attenuated lung injury as well as the bacterial load in infected lungs and in the blood (Figures 4K–N). Similarly, the administration of additional recombinant IL-17A significantly attenuated systemic inflammation and extrapulmonary injury (Figures 4O, P), as evidenced by the downregulation of IL-6, TNF-α, and IL-1β, as well as the levels of biochemical markers of hepatic and renal function (AST, ALT, BUN, and CREA). The above results suggested that the host cannot produce sufficient IL-17A during PmA infection and that the Th17 cell effector IL-17A effectively limits PmA infection.

### STAT3 is critical for Th17 cell development during *Pasteurella multocida* serotype A infection

**Figure 5:**
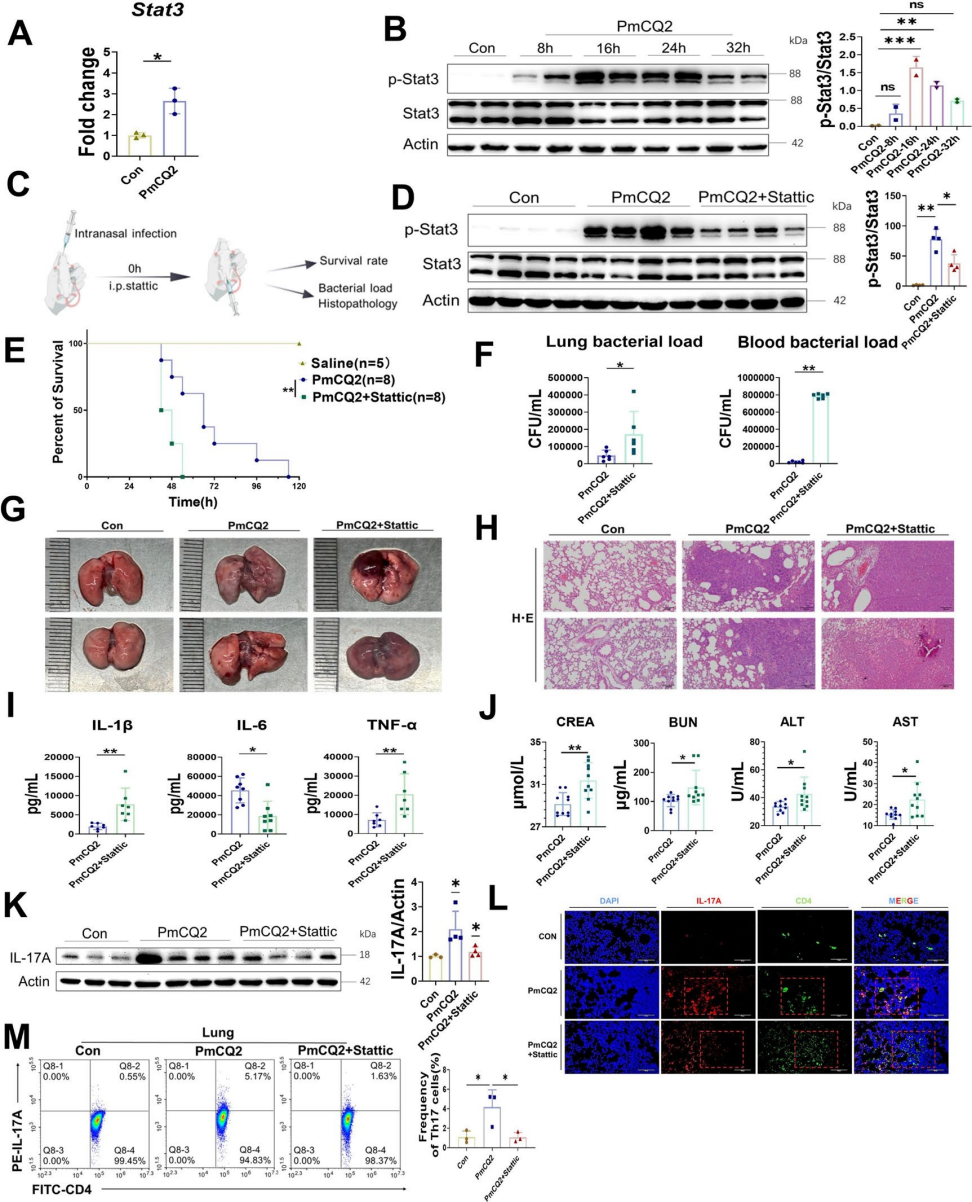
**STAT3 is critical for Th17 cell development during *Pasteurella multocida*** **serotype A infection.** **A** Transcription levels of Stat3 in PmCQ2-infected murine lungs at 24 hpi. **B** Western blot analysis and quantification of p-Stat3 and Stat3 in PmCQ2-infected murine lungs. **C** A scheme presents the Stattic treatment assay protocol. **D** Western blot analysis and quantification of p-Stat3 and Stat3 in PmCQ2-infected murine lungs. **E** Survival curves of PmCQ2-infected mice treated with or without 15 mg/kg Stattic. **F** The bacterial load of the infected lungs and blood at 32 hpi. **G** Photographs of murine lungs infected with PmCQ2 plus vehicle or 15 mg/kg Stattic at 32 hpi. **H** Representative images of HE staining of murine lungs infected with PmCQ2 plus vehicle or 15 mg/kg Stattic at 32 hpi. Scale bar = 200 μm. **I** Quantification of IL-6, TNF-α, and IL-1β PmCQ2-infected murine serum at 32 hpi. **J** Quantification of AST, ALT, BUN, and CREA levels in PmCQ2-infected murine serum at 32 hpi. **K** Western blot analysis and quantification of IL-17A in PmCQ2-infected murine lungs. **L** Representative images of mIHC staining of IL-17A and CD4 in Con- and PmCQ2-infected murine lungs at 32 hpi. The double-positive cells are mature Th17 cells. Scale bar = 100 μm. **M** Flow cytometry analysis and quantification of Th17 PmCQ2-infected murine lungs at 32 hpi. Every point represents one individual. *p < 0.05, **p < 0.01, ***p < 0.001.

As shown by our transcriptome data, the JAK–STAT pathway was enriched in PmA-infected lungs (Figure 2D). STAT3 has been reported to be closely associated with Th17 cell development [27]. Stat3 expression was significantly increased at both the transcriptional and translational levels in infected-murine lungs (Figures 5A, B). To determine the role of Stat3 in PmA infection, we used Stattic, a selective inhibitor of Stat3, in vivo (Figure 5C). As shown in Figures 5D, E, Stattic effectively inhibited the expression of p-Stat3 in the lungs of the mice and increased the number of deaths of the PmA-infected mice. In addition, Stattic aggravated PmA-induced lung injury and increased the bacterial load in infected lungs and blood (Figures 5F–H). Correspondingly, Stattic treatment enhanced PmA-induced systemic inflammation and extrapulmonary injury (Figures 5I, J), as evidenced by the upregulation of IL-6, TNF-α, and IL-1β, as well as biochemical markers of hepatic and renal function (AST, ALT, BUN, and CREA). In addition, multiple immunohistochemical and flow cytometric assays revealed that the inhibition of p-Stat3 limited Th17 cell development during PmA infection (Figures 5K–M). Taken together, these results suggested that STAT3 is critical for Th17 cell development in *P. multocida* infection.

### Regulation of Th17 cell/IL-17A activation via the IL-6–JAK2–STAT3 axis in *Pasteurella multocida* serotype A infection

**Figure 6:**
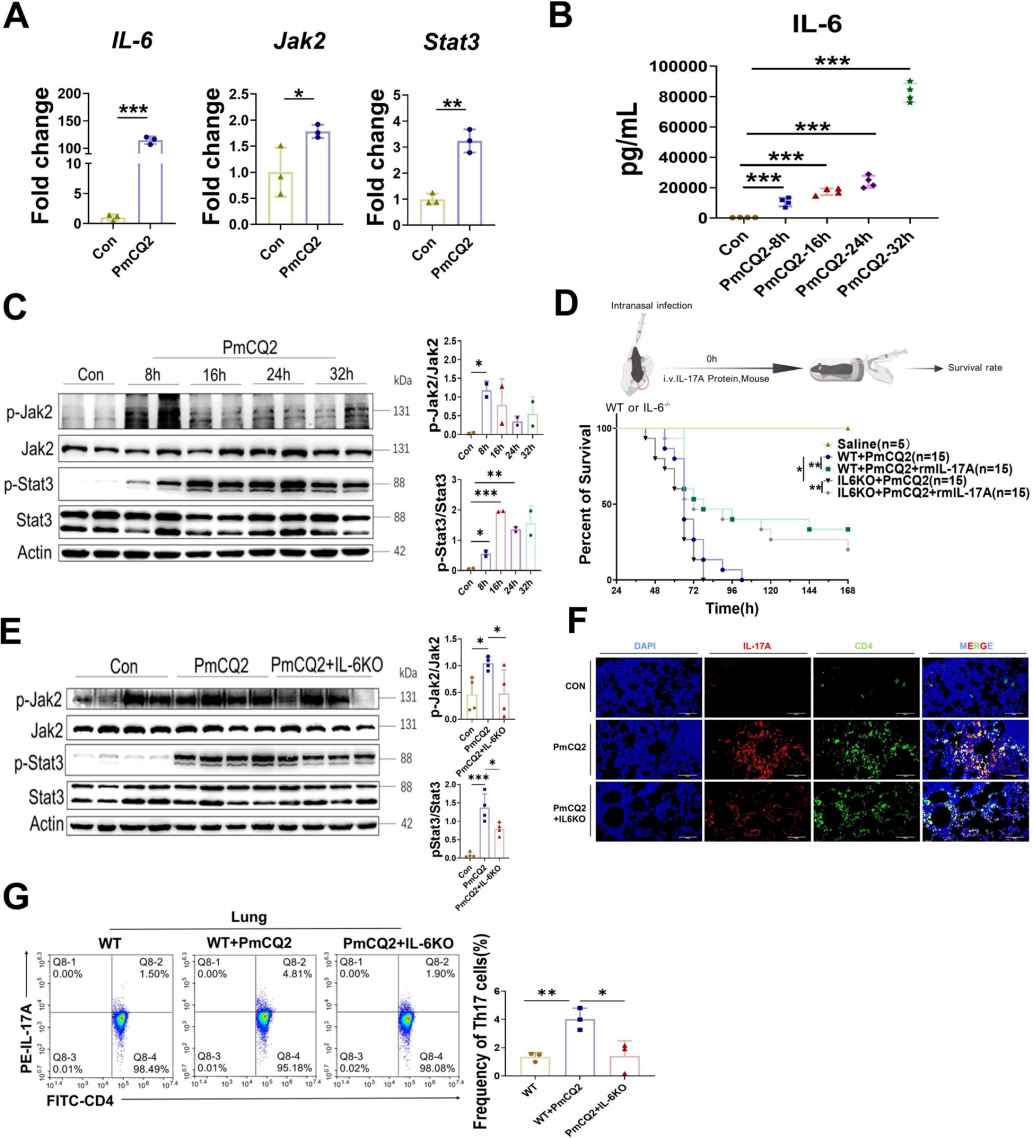
**Regulation of Th17 cell/IL-17A activation via the IL-6–JAK2–STAT3 axis in *Pasteurella multocida* serotype A infection.** **A** Transcription levels of IL-6, Jak2, and Stat3 in PmCQ2-infected murine lungs at 24 hpi. **B** Quantification of IL-6 in PmCQ2-infected murine serum in a time-dependent manner. **C** Western blot analysis and quantification of p-Jak2, Jak2, p-Stat3, and Stat3 in PmCQ2-infected murine lungs in a time-dependent manner. **D** A schematic of the IL-6-KO mouse treatment protocol at the top. The survival curves of PmCQ2-infected IL-6-KO mice treated with or without 10 μg/kg recombinant mouse IL-17A are shown. **E** Western blot analysis and quantification of p-Jak2, Jak2, p-Stat3, and Stat3 in PmCQ2-infected murine lungs. **F** Representative images of mIHC staining of IL-17A and CD4 in Con- and PmCQ2-infected murine lungs at 32 hpi. The double-positive cells are mature Th17 cells. Scale bar = 100 μm. **G** Flow cytometry analysis and quantification of Th17 PmCQ2-infected murine lungs at 32 hpi. Every point represents one individual. *p < 0.05, **p < 0.01, ***p < 0.001.

IL-6 has been reported to be an indispensable factor for Th17 cell development [27]. We detected significant increases in the levels of IL-6, Jak2, and Stat3 in the lungs of PmA-infected mice in the transcriptome (Figure 6A). The ELISA results also revealed an increase in IL-6 in PmA-infected mice (Figure 6B). Western blotting revealed that PmA infection indeed activated the Jak2–Stat3 pathway in vivo (Figure 6C), suggesting that the IL-6–JAK2–Stat3 axis may be involved in the development of Th17 cells during PmA infection. To further demonstrate that the IL-6–Jak2–Stat3 axis is involved in Th17 cell development during PmA infection, we used IL-6-KO mice and recombinant IL-17A. As shown in Figure 6D, IL-6-KO enhanced PmA-induced mouse death, whereas recombinant IL-17A rescued it. Western blot analysis revealed that IL-6-KO inhibited the expression of p-Jak2 and p-Stat3 (Figure 6E). In addition, multiple immunohistochemistry and flow cytometry experiments revealed that IL-6-KO significantly inhibited Th17 cell development and IL-17A secretion (Figures 6F, G). The above results suggested that the IL-6–JAK2–STAT3 axis is deeply involved in the activation of Th17 cells/IL-17A during PmA infection.

### IL-17A has potential for clinical application

**Figure 7:**
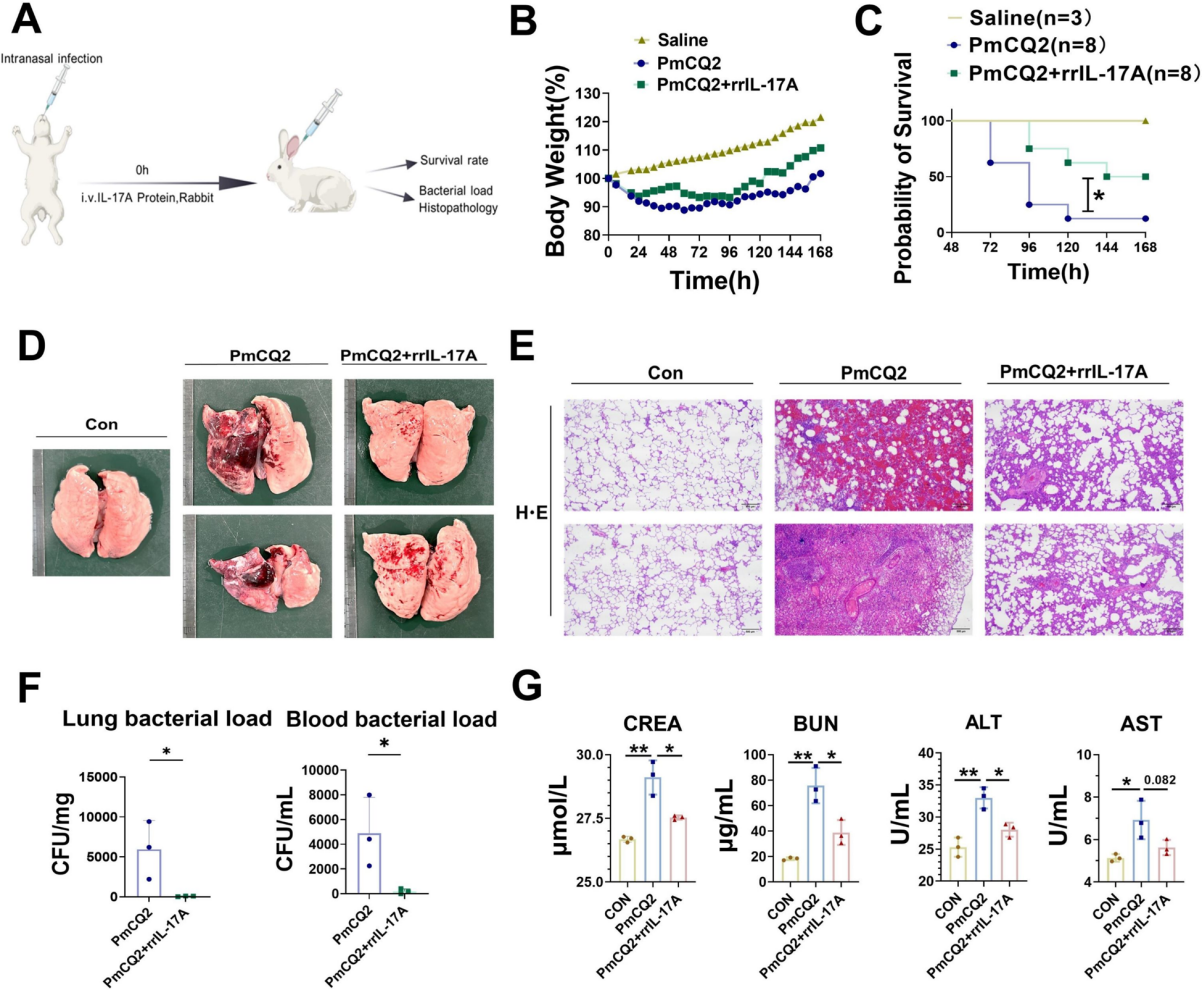
**IL-17A has potential for clinical application**. **A** Scheme showing the recombinant rabbit IL-17A treatment protocol. **B** Body weight (%) of PmCQ2-infected rabbits in different groups. **C** Survival curves of PmCQ2-infected rabbits treated with or without 5 μg/kg recombinant rabbit IL-17A. **D** Photographs of PmCQ2-infected rabbit lungs with or without 5 μg/kg recombinant rabbit IL-17A at 48 hpi. **E** Representative images of HE staining of PmCQ2-infected rabbit lungs with or without 5 μg/kg recombinant rabbit IL-17A at 48 hpi. Scale bar = 500 μm. **F** The bacterial load of the infected lungs and blood at 48 hpi. **G** Quantification of AST, ALT, BUN, and CREA levels in PmCQ2-infected rabbit serum at 48 hpi. Every point represents one individual. *p < 0.05, **p < 0.01.

**Figure 8:**
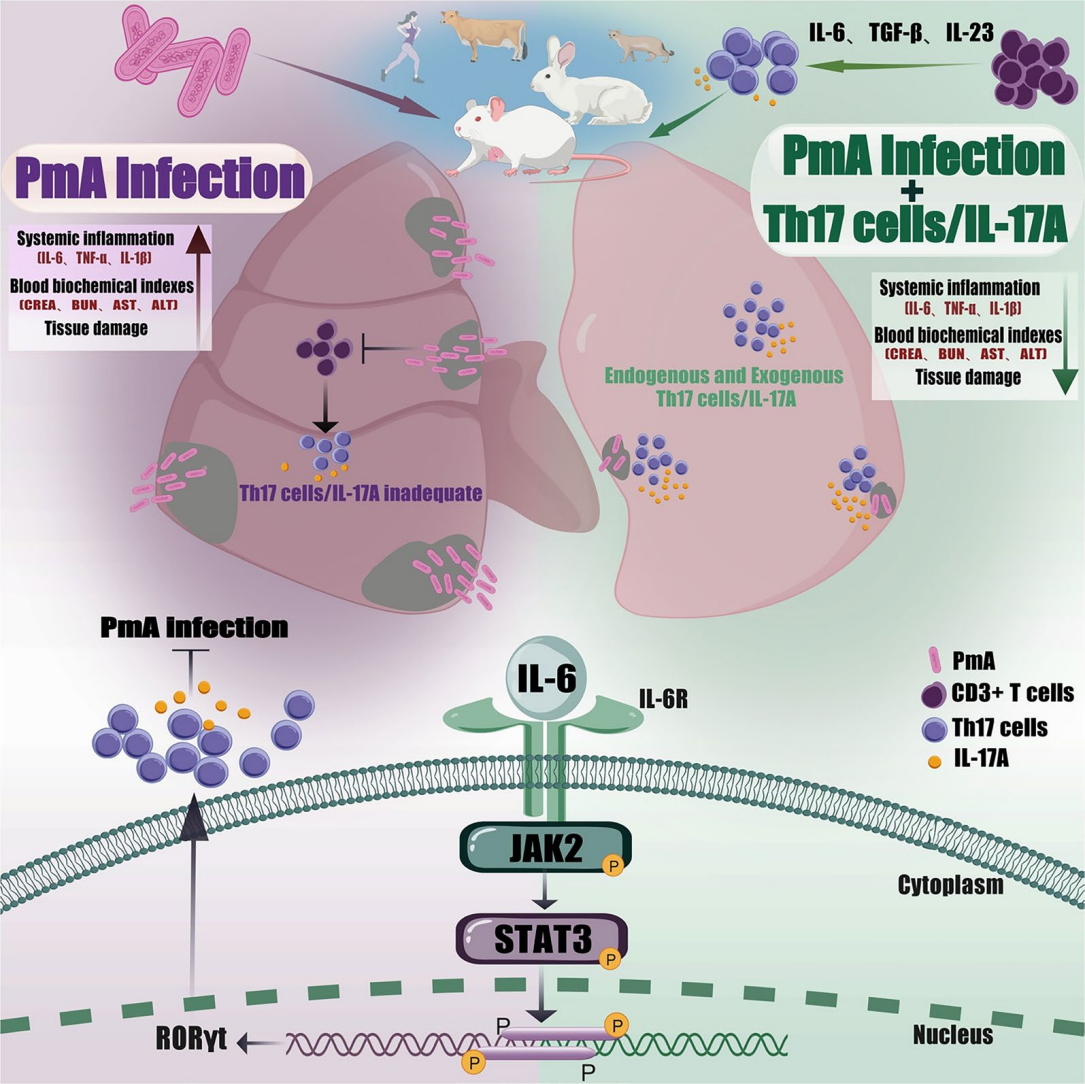
**Schematic showing that Th17 cells/IL-17A shape *Pasteurella multocida*** **serotype A infection.** Serotype A *Pasteurella multocida* (PmA) causes systemic infection and excessive release of inflammatory factors by destroying the lung barrier. In contrast to previous reports that PmA induces an excessive immune response, although PmA can induce a Th17 cell response, it inhibits T-cell immunity, resulting in insufficient activation of Th17 cells and their effector molecule IL-17A, which ultimately limits the clearance of PmA. At the molecular level, the IL-6–JAK2–STAT3 axis is strongly involved in Th17 cell/IL-17A activation during PmA infection. Importantly, targeting Th17 cells and IL-17A has potential for clinical application, as evidenced by the significant attenuation of PmA-induced lung injury and systemic inflammation as well as the reduction in animal mortality.

To further determine whether Th17 cells/IL-17A have potential clinical applications, we tested recombinant IL-17A in rabbits, a clinical animal model (Figure 7A). As shown in Figure 7B, the duration and magnitude of weight loss were significantly shorter in recombinant IL-17A-treated rabbits than in rabbits infected with PmCQ2 alone. Consistent with the results of the mouse experiments, recombinant IL-17A also significantly reduced the bacterial load, pulmonary and extrapulmonary injury (as evidenced by reduced levels of AST, ALT, BUN, and CREA), and animal death (Figures 7C–G). These results suggested that the development of relevant drugs targeting Th17 cells/IL-17A may be of clinical relevance. A hypothetical plot is shown in Figure 8.

## Discussion

*Pasteurella multocida* (Pm) is a Gram-negative respiratory pathogen with a wide range of hosts including mice, rats, rabbits, cattle, sheep, and humans [28–30]. Owing to its high lethality in domestic animals, Pm causes significant economic losses worldwide. Remarkably, a growing number of studies (including our own) have found that highly lethal *Pasteurella multocida* serotype A (PmA) causes not only severe respiratory damage but also a range of extrapulmonary infections through the bloodstream [4, 31, 32]. In other words, the inability of the host to generate sufficient antimicrobial immunity in the lungs leads to the breakthrough of the pulmonary barrier by PmA into peripheral tissues. In a previous study, we first demonstrated that PmA-induced bacteremia is an important pathogenic mechanism leading to host death [4]. Therefore, we believed that immunity must be mobilized to control the initial stages of lung infection.

To understand the immune environment of the lungs after PmA infection, we performed RNA sequencing (RNA-seq) and found a significant enrichment of Th17-cell-associated signals. These results are consistent with our previous report [15]. To date, no study has explored the specific role of Th17 cell in PmA infection. However, in the past, researchers have suggested that the excessive immune-inflammatory response induced by PmA is a key factor leading to host death. For example, 18β-glycyrrhizinic acid alleviates Pm infection by attenuating vascular inflammatory damage [33]. Melatonergic signaling inhibits PmA infection by limiting interleukin-1β-dependent inflammation [34]. M1 macrophages were found to be pro-inflammatory cells that predominate in PmA-infected lungs [10]. Similarly, we ourselves have found that PmA causes host death by inducing a cytokine storm [4]. As a result, researchers have routinely considered the inflammatory response to be bad in PmA infection and have been searching for anti-inflammatory methods to control PmA, ignoring the possibility of an immune deficiency in the lungs.

In the current work, we noted that PmA infection limited T-cell immunity in mammals, as evidenced by reduced CD3^+^ T-cell expression, and we are exploring the mechanisms behind this. We further demonstrated that Th17 cell/IL-17A activation was also affected, and that they play a key protective role against PmA infection, as supplementation with additional Th17 cells/IL-17A significantly reduced lung bacterial load and lung infections, and improved survival in vivo. Our findings suggested that during PmA infection, while overall inflammatory factors and immune responses may be excessive, certain individual immune and inflammatory factors are deficient. Currently, there are some evidence to support our results. Pm has been reported to reduce lymphocyte numbers through apoptosis or cytolysis [11, 12]. *Pasteurella multocida* toxin is thought to manipulate T-cell differentiation [13]. PM27/UPase purified from Pm showed significant antiproliferative effects on Concanavalin-A-stimulated mouse spleen cells [14]. Recently, the lipoproteins of Pm have been reported to have an immune evasion ability [35]. In addition, Th17 cells/IL-17A have been reported to have a lethal effect on extracellular bacteria, of which Pm is a typical type. If PmA-induced hyperinflammation is viewed as a harsh symphony, then we believe that it is better to adjust the performance of the instrument (clarifying and adjusting the roles of immune cells and inflammatory factors) than to reduce the volume (lowering the overall inflammatory response). Therefore, we believe that our findings to control PmA infection by identifying factors that contribute to host immunodeficiency will provide new insights for future studies.

In the present study, we found a significant reduction in bacterial load after supplementation with additional Th17 cells/IL-17A. One possible explanation is that IL-17A synergistically activates macrophages with interferon (IFN)-γ, which enhances the expression of inducible nitric oxide synthase (iNOS) and promotes the release of nitric oxide (NO), which plays a crucial role in bacterial clearance [36]. In addition, IL-17A binds to its receptor IL-17RA, which then activates a variety of downstream pathways, including antimicrobial peptides, pro-inflammatory cytokines, and chemokines [37]. The mechanism of Th17 cell/IL-17A against PmA infection needs to be further elucidated by us in the future. To understand the molecular mechanism of PmA activation of Th17 cell and IL-17A, we used IL-6-KO mice. IL-6 plays a decisive role in the maturation of Th17 cells [19]. IL-6-KO mice also show increased susceptibility to most pathogens, such as herpes simplex virus and *Mycobacterium tuberculosis* [38–40]. In the current study, we found that PmA-infected IL-6-KO mice had significantly increased mortality and decreased p-Jak2, p-Stat3, Th17 cells, and IL-17A. Exogenous IL-17A antagonized the increased mortality, suggesting that IL-6 is important for Th17 cell/IL-17A activation during PmA infection. It is well known that the IL-6 downstream factor STAT3 is essential for Th17 cell/IL-17A activation [41], and we have also found that pharmacological inhibition of Stat3 made mice more sensitive to PmA. These data suggested that the IL-6–Jak2–Stat3 axis was important for Th17 cell/IL-17A activation during PmA infection. Indeed, combining the results of previous studies and the present study, we found that the correlation between the expression of p-Jak2 and the expression of IL-6 was not strong during PmA infection (especially 8 h post-infection), suggesting that PmA may impair IL-6–Jak2 signaling, thereby inhibiting the activation of Th17 cell/IL-17A and achieving immune escape. We will explore the exact mechanisms in subsequent studies.

In conclusion, our current study revealed that Th17 cell/IL-17A is critical in the fight against PmA and that the IL-6–Jak2–Stat3 axis promoted Th17 cell/IL-17A activation during PmA infection in vivo. In addition, our findings provided valuable insights for future PmA studies that aim to control PmA infection by identifying factors that contribute to host immunodeficiency, rather than following the rules to suppress inflammatory or immune responses. Importantly, our findings suggested that targeting the Th17 cell/IL-17A activation pathway may be a potential strategy for the treatment of pasteurellosis and other respiratory bacterial diseases.

## Supplementary Information

**Additional file 1**: **On the top are representative images of mIHC staining of IL-17A and CD4 in rabbit lungs at 36 hpi.** Scale bar = 100 μm. On the bottom is the flow cytometry analysis and quantification of Th17 cells in Con- and PmCQ2-infected rabbit lungs at 36 hpi.

**Additional file 2**: **Flow cytometry analysis and quantification of CD3+ cells in Con- and PmCQ2-infected rabbit lungs at 36 hpi.**

**Additional file 3**: **Gating strategy for the identification of Th17 cells in this study and flow cytometry analysis and quantification of Th17 cells in our in vitro culture.**

**Additional file 4**: **Identification of IL-6-KO mice via PCR.**

**Additional file 5**: **Primer list.**

**Additional file 6**: **Raw RNA-seq data used in the study.**

## Data Availability

The data used to support the findings of this study are available from the corresponding author upon reasonable request.
