# Supplementary figures and images for: Th17 cells/IL-17A shape Pasteurella multocida serotype A infection in murine and rabbit models
Source: Vet Res. 2025 Dec 16;56:229. doi: 10.1186/s13567-025-01662-1 (PMC12707012; doi:10.1186/s13567-025-01662-1)

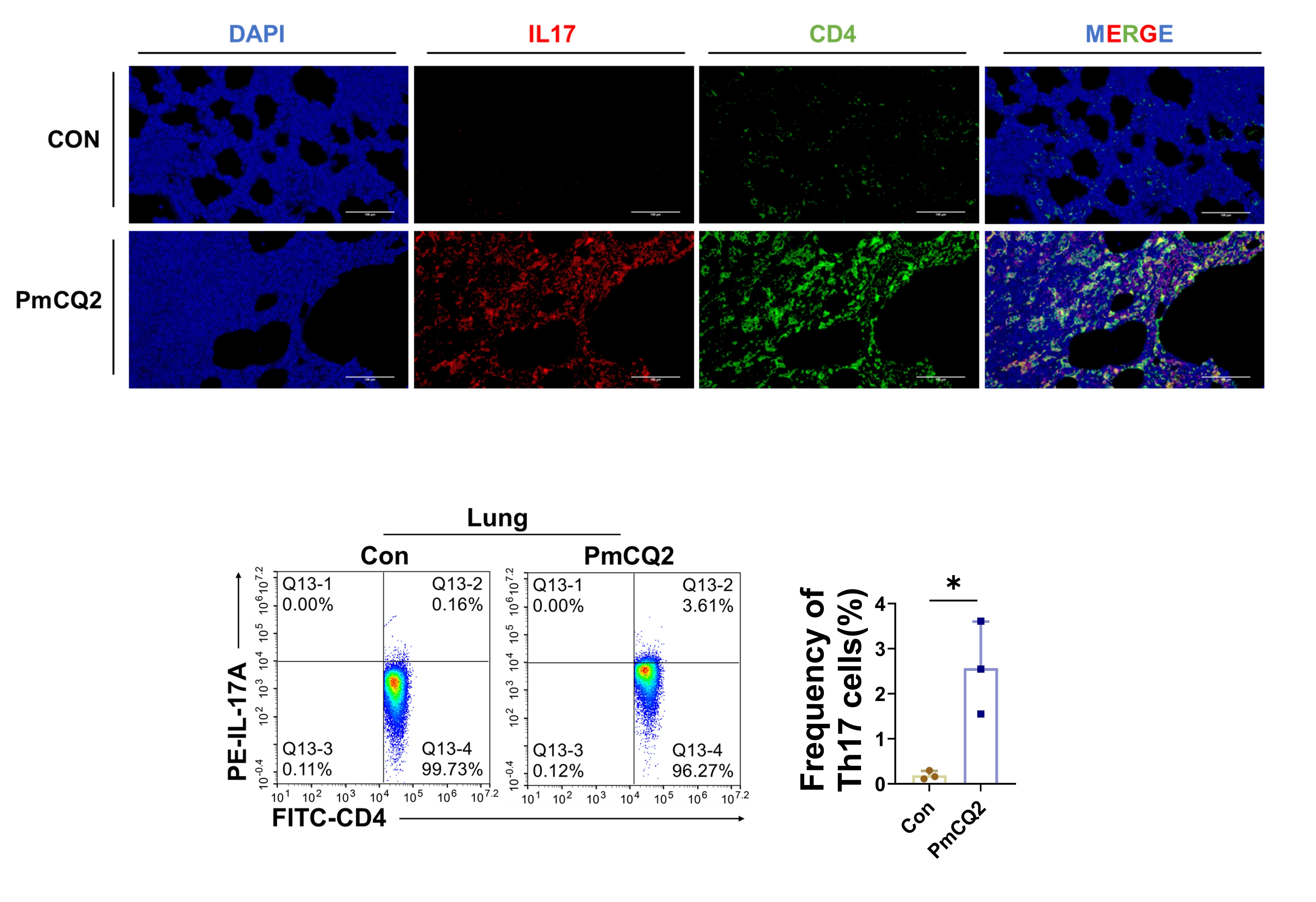

Supplement: Supplementary file 1 — Additional file 1: On the top are representative images of mIHC staining of IL-17A and CD4 in rabbit lungs at 36 hpi. Scale bar = 100 μm. On the bottom is the flow cytometry analysis and quantification of Th17 cells in Con- and PmCQ2-infected rabbit lungs at 36 hpi. [file 13567_2025_1662_MOESM1_ESM.jpg]

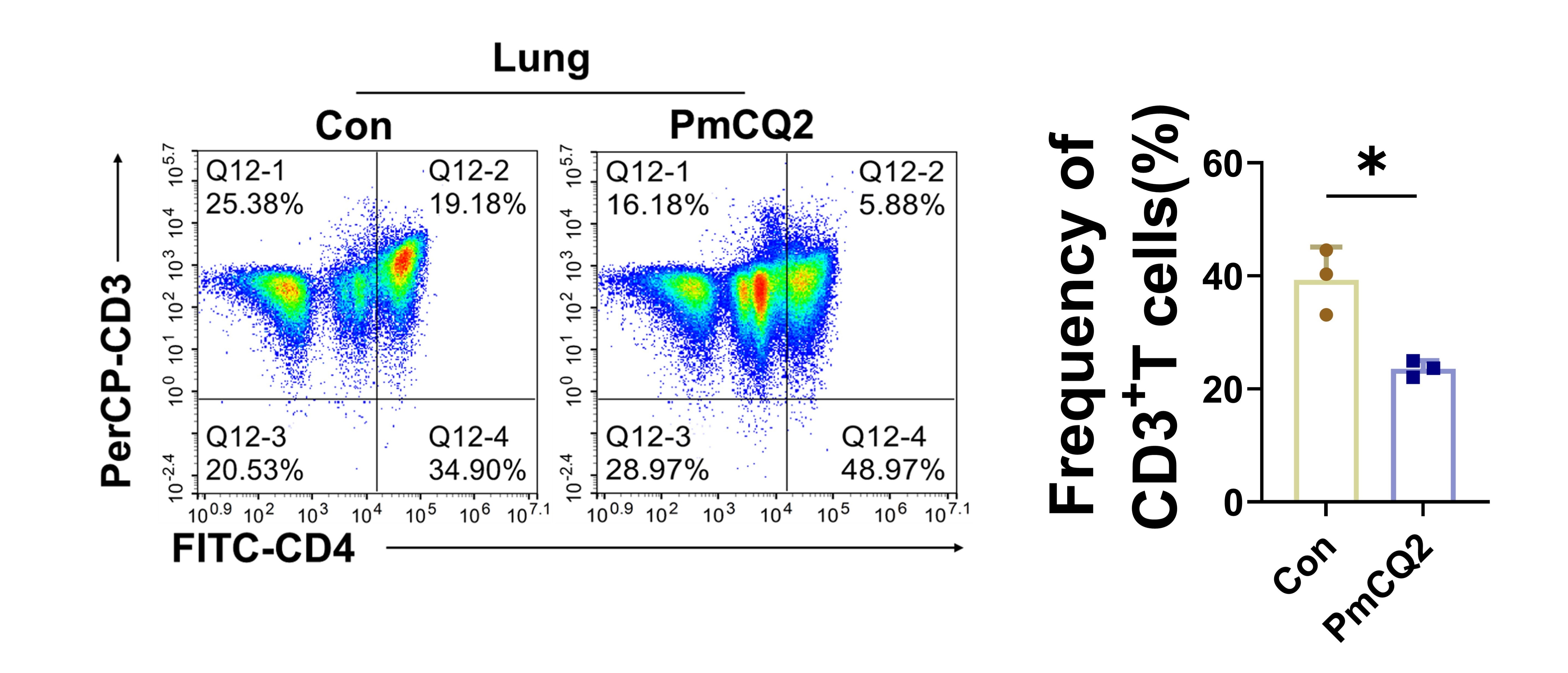

Supplement: Supplementary file 2 — Additional file 2: Flow cytometry analysis and quantification of CD3+ cells in Con- and PmCQ2-infected rabbit lungs at 36 hpi. [file 13567_2025_1662_MOESM2_ESM.jpg]

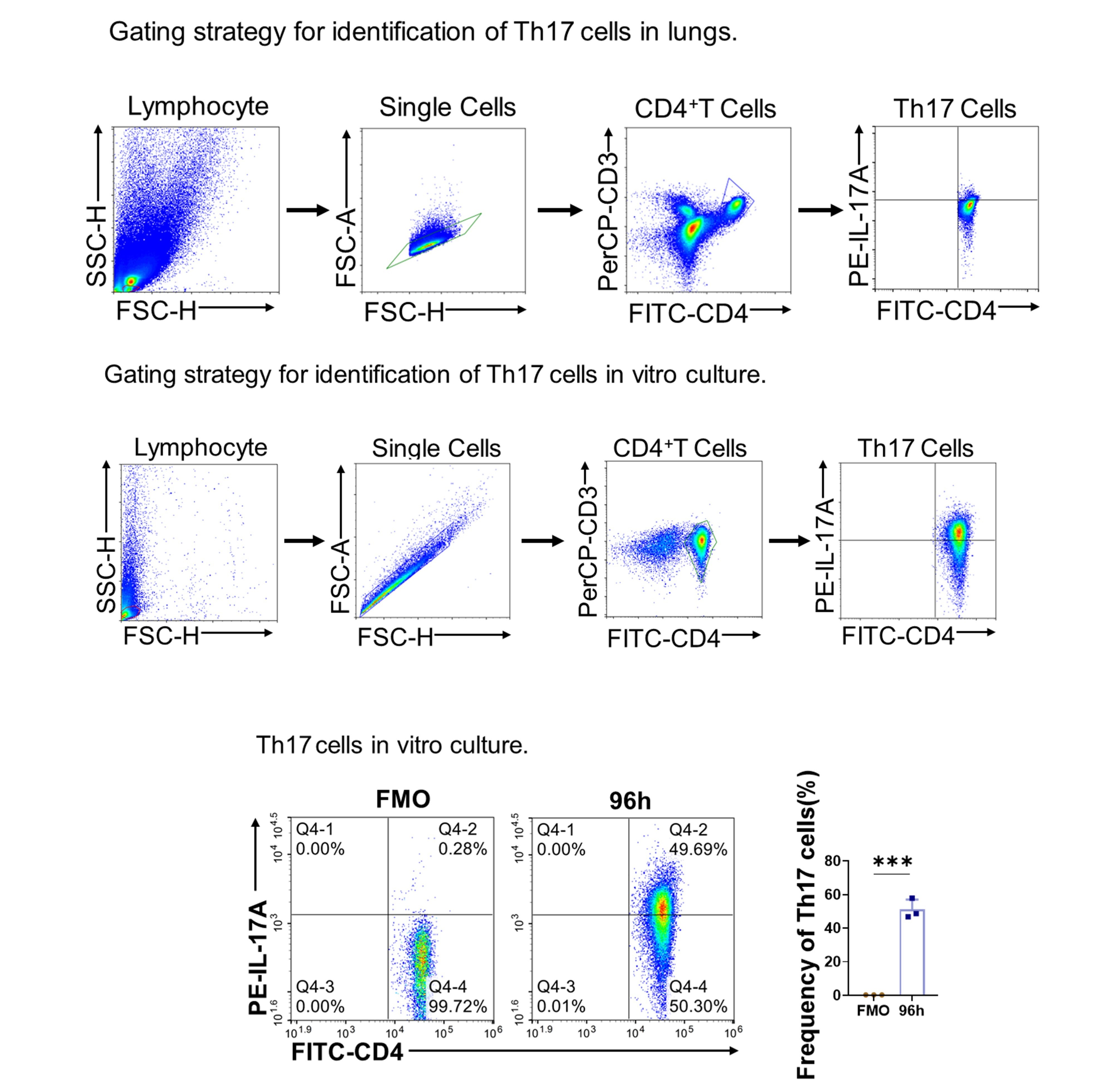

Supplement: Supplementary file 3 — Additional file 3: Gating strategy for the identification of Th17 cells in this study and flow cytometry analysis and quantification of Th17 cells in our in vitro culture. [file 13567_2025_1662_MOESM3_ESM.jpg]

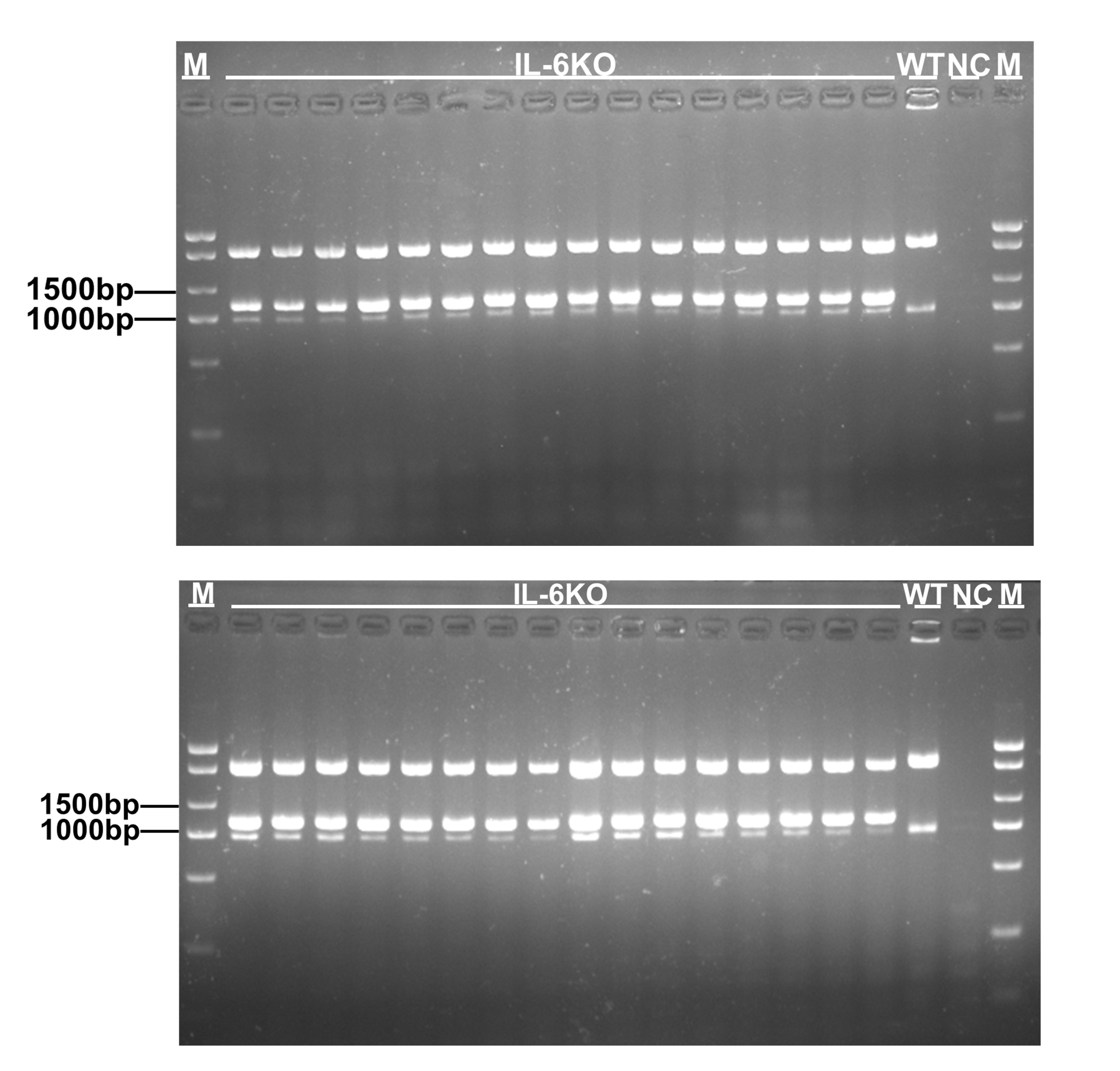

Supplement: Supplementary file 4 — Additional file 4: Identification of IL-6-KO mice via PCR. [file 13567_2025_1662_MOESM4_ESM.jpg]
